# Single-cell RNA sequencing of intestinal Behçet’s disease identifies putative pathogenic programs and potential therapeutic targets

**DOI:** 10.17305/bb.2026.14124

**Published:** 2026-05-06

**Authors:** Chengzhen Lyu, Kun He, Shuai Li, Ziqi Guo, Xiaxiao Yan, Rou Tang, Yanan Shi, Jianyekai Tuerheng, Weiyang Zheng, Lingjuan Jiang, Yi Hu, Dong Wu

**Affiliations:** 1 Department of Gastroenterology, State Key Laboratory of Complex Severe and Rare Diseases, Peking Union Medical College Hospital, Chinese Academy of Medical Sciences and Peking Union Medical College, Beijing, China; 2 Chinese Academy of Medical Sciences and Peking Union Medical College, Beijing, China; 3 Emergency Department, State Key Laboratory of Complex Severe and Rare Diseases, Peking Union Medical College Hospital, Chinese Academy of Medical Sciences and Peking Union Medical College, Beijing, China; 4 Department of Pharmacy, Peking Union Medical College Hospital, Chinese Academy of Medical Sciences and Peking Union Medical College, Beijing, China; 5 State Key Laboratory of Complex Severe and Rare Diseases, Biomedical Engineering Facility of National Infrastructures for Translational Medicine, Peking Union Medical College Hospital, Chinese Academy of Medical Sciences and Peking Union Medical College, Beijing, China; 6 Biomarker Discovery and Validation Facility, Institute of Clinical Medicine, Peking Union Medical College Hospital, Chinese Academy of Medical Sciences and Peking Union Medical College, Beijing, China

**Keywords:** Behçet syndrome, single-cell RNA sequencing, Crohn disease, intestinal mucosa, cellular heterogeneity, inflammation, biological markers, therapeutic targets

## Abstract

Intestinal Behçet’s disease is a rare, refractory subtype of systemic vasculitis, characterized by deep ileocecal ulcers and extensive clinical overlap with Crohn’s disease, while the cellular and molecular mechanisms underlying its pathogenesis remain poorly characterized. This study aimed to delineate the single-cell transcriptomic landscape of intestinal Behçet’s disease, characterize its putative disease-associated transcriptomic signatures, and identify potential candidates for differential diagnosis and targeted treatment. We performed single-cell RNA sequencing on paired inflamed and histologically normal terminal ileum biopsy specimens from 4 patients with active intestinal Behçet’s disease, and integrated our dataset with a public single-cell dataset from 12 Crohn’s disease patients and 6 healthy controls for systematic bioinformatic analysis. We thereby constructed the first single-cell transcriptomic atlas of human intestinal Behçet’s disease, profiling 98,119 high-quality cells to identify 3 major cell lineages, 20 distinct cell populations, and 41 functionally defined cell subtypes. Our analysis indicated that intestinal Behçet’s disease may be characterized by robust stromal compartment activation, extracellular matrix remodeling, and potentially distinct epithelial antimicrobial signatures, which showed notable differences from the prominent epithelial barrier dysfunction and interferon-driven immune activation observed in Crohn’s disease in this parallel intra-disease comparison framework. We further identified predicted pathogenic crosstalk between endothelial cells and neutrophils, which may be mediated by collagen/laminin-CD44 axes. Our findings thereby characterize the potential pathogenic features of intestinal Behçet’s disease, and provide hypothesis-generating clues for the clinical management of this rare disorder.

## Introduction

Behçet’s disease (BD) is a chronic, multisystemic vasculitis of unknown etiology, characterized by recurrent oral ulcers, genital lesions, ocular inflammation, and gastrointestinal involvement [1]. Intestinal BD, a rare and refractory subtype affecting 15%–20% of BD patients, presents with deep penetrating ileocecal ulcers and is associated with high complication rates and poor clinical outcomes [2, 3]. A significant clinical challenge arises from the substantial phenotypic overlap between intestinal BD and Crohn’s disease (CD), the most prevalent subtype of inflammatory bowel disease. This overlap often results in diagnostic delays and ongoing uncertainty regarding whether these conditions are distinct pathogenic entities or overlapping clinical phenotypes [4, 5]. Currently, the cellular and molecular mechanisms driving intestinal BD remain poorly understood, which severely hampers the development of targeted diagnostics and therapies.

Recent advances in single-cell RNA sequencing (scRNA-seq) have revolutionized research in human diseases by enabling high-resolution dissection of cellular heterogeneity, cell-type-specific pathogenic programs, and intercellular communication networks [6]. These technologies have provided critical pathogenic insights across a wide range of inflammatory and malignant disorders, even with limited input tissue, making them particularly relevant for rare diseases like intestinal BD. Although scRNA-seq has been applied to peripheral blood and aortic tissues from BD patients [7–9], no studies have yet profiled intestinal lesions from BD patients at single-cell resolution, nor has a systematic comparative analysis with CD been conducted [10–12].

In this study, we performed scRNA-seq on paired inflamed and histologically normal terminal ileum biopsies from patients with active intestinal BD, integrating our data with a publicly available scRNA-seq dataset of CD and healthy control (HC) tissues. We hypothesized that intestinal BD possesses a unique cellular and molecular landscape distinct from CD, with core pathogenic programs reflecting its underlying vasculitic origin. The primary objectives of this study were to construct the first single-cell transcriptomic atlas of intestinal BD, delineate its putative disease-associated cellular and signaling signatures, map inferred dysregulated intercellular communication networks, and identify potential diagnostic biomarkers and therapeutic targets. Our findings suggest that intestinal BD may be a stromal-vascular-dominant inflammatory disorder with distinct transcriptomic features compared to CD, providing a foundational resource for precision medicine development for this rare disease.

## Materials and methods

### Study participants and sample collection

Patients with active intestinal BD who met both the 1990 International Study Group criteria and the 2014 International Criteria for BD were recruited from Peking Union Medical College Hospital [13, 14]. Eligible patients were aged 20–65 years, with endoscopically and histologically confirmed gastrointestinal involvement, and no confounding systemic or intestinal disorders. A total of four intestinal BD patients were enrolled between March and December 2025, with detailed clinical characteristics provided in Table S1. Paired inflamed terminal ileum biopsy specimens and histologically normal adjacent tissues (sampled 5 cm away from the ulcer margin) were collected from each patient via endoscopic biopsy [15, 16].

### Single-cell suspension preparation, library construction, and sequencing

Freshly collected biopsy tissues were immediately processed for single-cell suspension preparation. Tissues were washed twice with pre-chilled Roswell Park Memorial Institute 1640 medium (RPMI 1640) supplemented with 0.04% bovine serum albumin (BSA), minced into approximately 0.5 mm^3^ fragments, and digested in freshly prepared enzymatic solution at 37^∘^C for 30–60 min, with gentle inversion every 5–10 min. The digested suspension was filtered through a 40 µm cell strainer to remove tissue debris, followed by centrifugation at 300×g for 5 min at 4^∘^C. Red blood cell (RBC) lysis was performed using RBC lysis buffer (magnetic cell separation (MACS), Catalog No. 130-094-183) at 4^∘^C for 10 min, followed by two additional washes with RPMI 1640 medium. Cell concentration and viability were quantified using a Luna Cell Counter. Only suspensions with viability > 80% were used for library construction. All eight collected samples (four paired patients) met quality criteria; no samples were excluded.

Single-cell library construction was performed using the 10× Genomics Chromium Next Gel Bead-in-emulsion (GEM) Single Cell 3' Reagent Kits v3.1 (Cat No. PN1000268), following the manufacturer’s protocol, with a target cell recovery of 10,000 cells per sample [17]. High-throughput sequencing was conducted on the Illumina X plus paired-end 150 bp sequencing (PE150) platform.

### Data preprocessing and quality control

Raw sequencing reads were processed using CellRanger v7.1.0 (10× Genomics) to align them against the Genome Reference Consortium Human Build 38 (GRCh38) and generate gene count matrices [18]. To ensure data purity, we employed CellBender v0.3.2 to remove background contamination [19]. We used scDblFinder v1.22.0 to identify and filter out doublets, excluding any cells with a doublet score > 0.3 [20]. Further quality control was performed in Scanpy v1.11.4 [21]. Specifically, we removed cells exhibiting total Unique Molecular Identifier (UMI) counts below 500 or above 30,000, fewer than 200 detected genes, or mitochondrial/hemoglobin gene proportions exceeding 20%. Given the high turnover of the intestinal epithelium characterized by continuous regeneration and apoptosis, as well as the necessity to retain biologically relevant cells, including those under inflammatory stress, we empirically set the mitochondrial gene proportion threshold at 20% for this analysis. This approach aligns with established protocols from previously published intestinal scRNA-seq studies, optimizing the balance between removing low-quality cells and retaining metabolically active functional cells [22–24]. Full sample-level quality control metrics are provided in Supplemental File 1.

### Data integration and cell annotation

**Figure 1.**
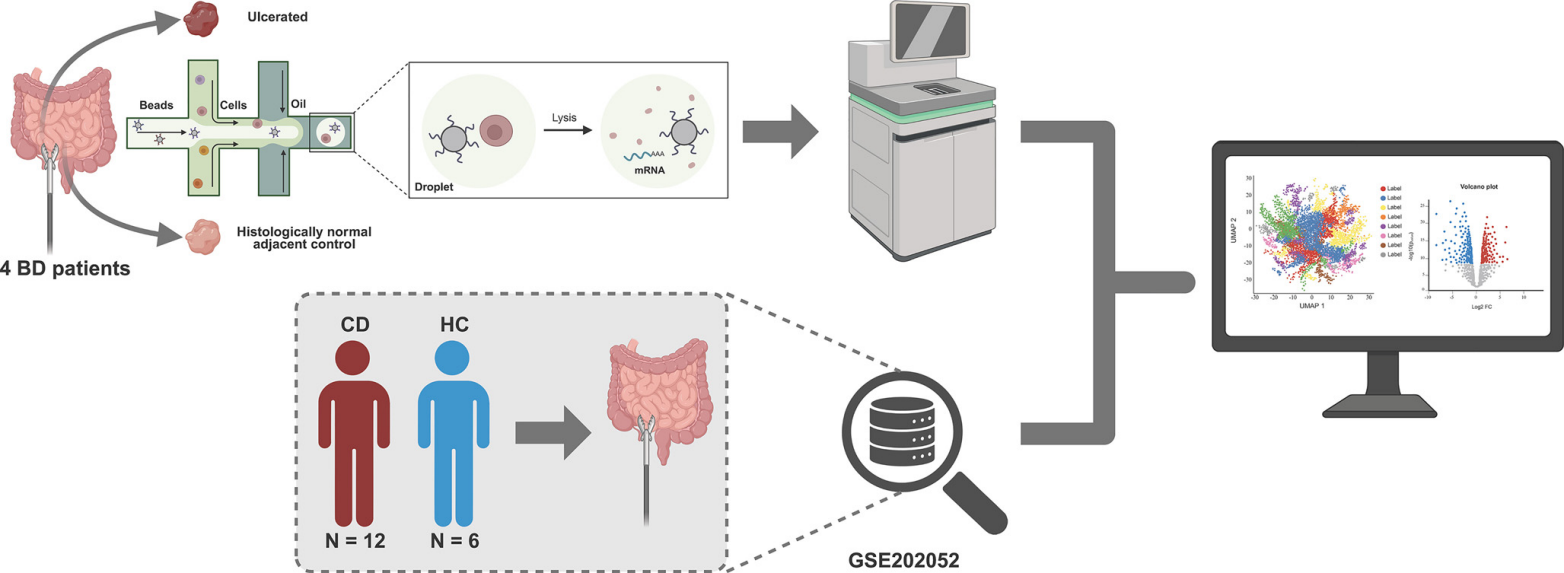
**Schematic overview of the experimental and analytical workflow.***Created in https://BioRender.com.* Abbreviations: BD, Behçet’s disease; CD, Crohn’s disease; HC, healthy control.

**Figure S1.**
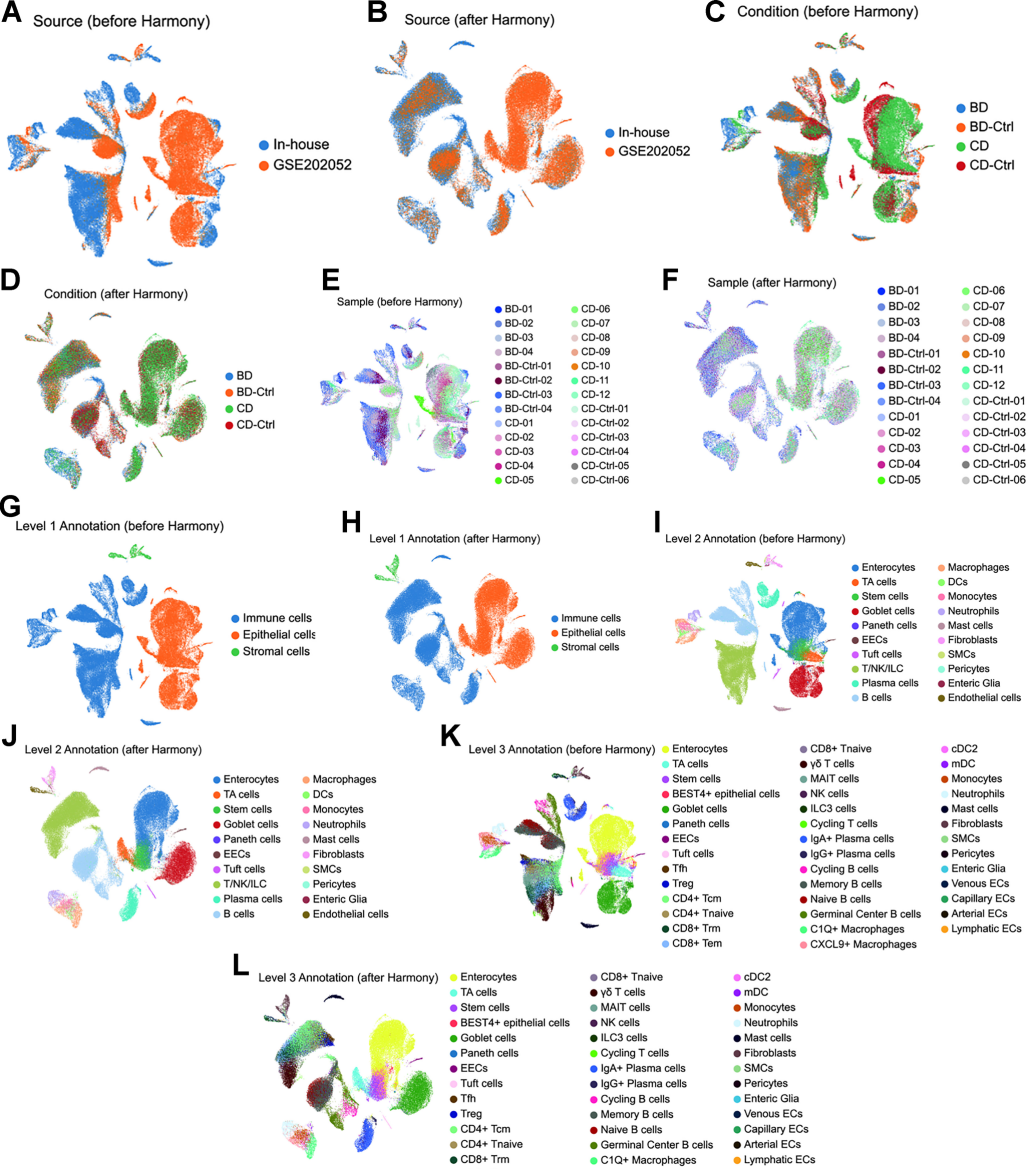
**Visual validation of dataset integration and batch-effect correction.** (A–F) UMAP embeddings of the single-cell datasets colored by data source (A) before and (B) after Harmony integration, by study condition (C) before and (D) after Harmony integration, and by sample origin (E) before and (F) after Harmony integration. (G–L) UMAP visualizations colored by the three-level hierarchical annotation strategy: Level 1 annotation (G) before and (H) after Harmony integration, Level 2 annotation (I) before and (J) after Harmony integration, and Level 3 annotation (K) before and (L) after Harmony integration. Abbreviations: BD, Behçet’s disease; CD, Crohn’s disease; UMAP, uniform manifold approximation and projection.

To enhance our analysis, we incorporated a publicly available scRNA-seq dataset (Gene Expression Omnibus (GEO) accession: GSE202052), which includes terminal ileum tissues from 12 patients with CD and 6 HCs (Figure 1) [22]. Batch effects across datasets and samples were corrected using harmonypy v0.0.10 [21]. Visual validation of successful batch-effect mitigation is presented in Figure S1, which provides side-by-side comparisons of uniform manifold approximation and projection (UMAP) embeddings before and after Harmony integration. To ensure rigorous comparison while avoiding the confounding effects of disparate control baselines, our analytical framework employed parallel intra-disease comparisons rather than direct inter-disease quantification. We first identified transcriptomic alterations within each disease cohort (BD: inflamed vs. adjacent uninflamed; CD: inflamed vs. HC) and subsequently cross-referenced the alteration profiles to identify BD-specific signatures. Normalization, scaling, highly variable gene (HVG) selection, UMAP, and Leiden clustering were performed using Scanpy v1.11.4 [25].

**Figure 2.**
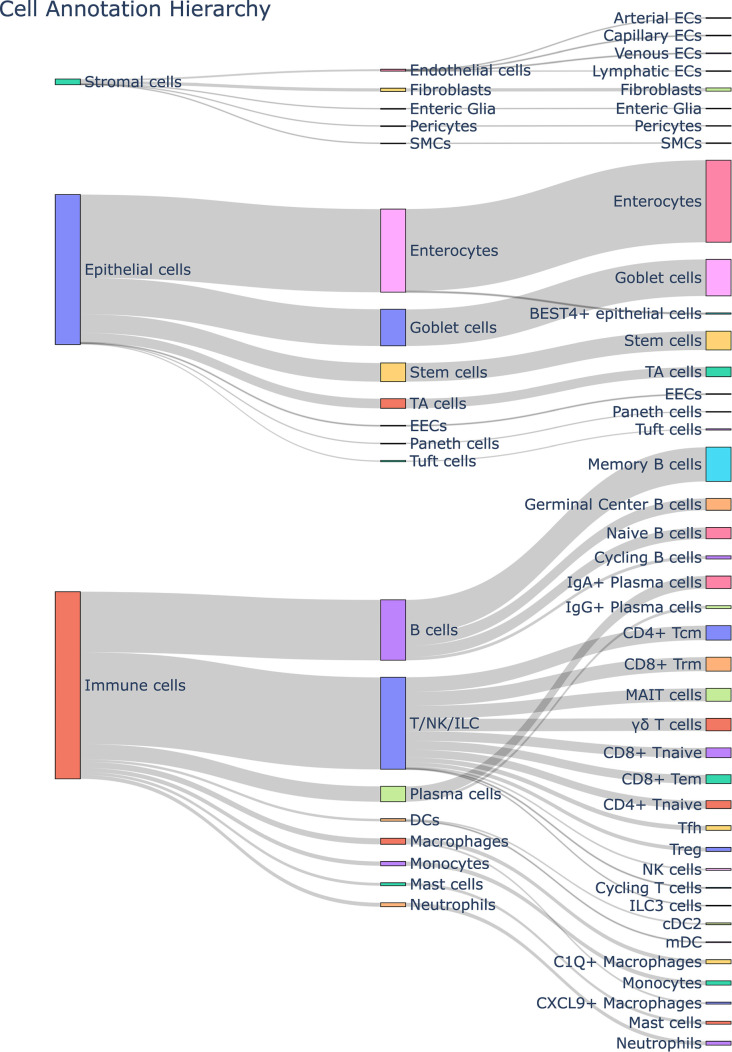
**Three-step hierarchical cell annotation strategy.** Abbreviations: B, B lymphocyte; CD, cluster of differentiation; cDC2, conventional dendritic cell type 2; CXCL, C-X-C motif chemokine ligand; DCs, dendritic cells; EC, endothelial cell; EECs, enteroendocrine cells; IgA, immunoglobulin A; IgG, immunoglobulin G; ILC, innate lymphoid cell; ILC3, innate lymphoid cell type 3; MAIT, mucosal-associated invariant T; mDC, myeloid dendritic cell; NK, natural killer; SMCs, smooth muscle cells; T, T lymphocyte; TA, transit amplifying; Tcm, central memory T; Tem, effector memory T; Tfh, T follicular helper; Tna, naive T; Treg, regulatory T; Trm, tissue-resident memory T.

We adopted a three-step hierarchical strategy to annotate cell identity (Figure 2). Firstly, we utilized canonical markers to partition all cells into three major lineages: immune cells (PTPRC, CD3D, CD79A), epithelial cells (EPCAM, KRT8, KRT18), and stromal cells (DCN, COL1A1, VIM) (Level 1). Next, we recursively sub-clustered each major lineage at a higher resolution, identifying 20 phenotypically distinct subpopulations (Level 2). Finally, further sub-clustering and manual curation based on well-established marker genes from Azimuth, CellMarker 2.0, and published literature resolved a total of 41 functionally defined cell subtypes (Level 3) [18, 23, 26]. Ratio of observed/expected (Ro/e) abundance analysis was conducted to quantify deviations in cellular composition between inflamed lesions and corresponding control samples [27]. Comprehensive quality control datasets across six hierarchical levels (source, condition, sample, Level 1 annotation, Level 2 annotation, and Level 3 annotation), including tables detailing the number of detected genes, total UMI counts, total and percentage of mitochondrial counts, total and percentage of ribosomal counts, and scDblFinder-derived doublet scores for quality control (QC)-passed cells included in the final analysis, are provided in Supplemental File 2.

### Differential expression and functional pathway analysis

At the single-cell level, we utilized the Wilcoxon rank-sum test to identify cell-type-specific differentially expressed genes (DEGs; adjusted *P* < 0.05 and |log2FoldChange| > 1) and applied Area Under the Curve-based cell scoring (AUCell) (executed via the decoupler.mt.aucell function with default parameters, leveraging relevant databases retrieved from https://omnipathdb.org/) to evaluate intrinsic pathway activation using Molecular Signatures Database (MSigDB) Hallmark gene sets, Gene Ontology Biological Process (GO BP) terms, and the Transcriptional Regulatory Relationships Unraveled by Sentence-based Text mining (TRRUST) transcription factor (TF) regulatory network [28–31]. Subsequent statistical comparisons were conducted using the rankby_group function (method = “*t*-test_overestim_var”). To ensure rigorous evaluation, differential comparisons were strictly limited to cell subpopulations containing a minimum of 10 cells in both the test and reference groups. We focused specifically on upregulated pathways within the test group (stat > 0), extracting the top five pathways for each group to formulate a non-redundant list for downstream visualization after the removal of duplicate terms. Concurrently, we constructed pseudobulk expression profiles by aggregating raw counts for any cell subpopulation containing at least three biological replicates (samples) per condition [32, 33]. These pseudobulk profiles were subsequently analyzed via DESeq2, employing tailored design formulas for distinct comparisons: ∼ Patient_ID + Condition for the BD vs BD-Ctrl comparison, and ∼ Condition for the CD vs CD-Ctrl comparison, to define robust sample-level DEGs, and the results were submitted to Gene Set Enrichment Analysis (GSEA) to capture global, condition-driven reactome shifts (using the Reactome pathway database) [34–36]. Additionally, TF activity inference was implemented using univariate linear model (ULM) activity scores with prior knowledge sourced from the Collection of Transcription Regulation Interactions (CollecTRI) gene regulatory network database and Pathway RespOnsive GENes (PROGENy) pathway activity database [37]. Methodologically, the scoring procedures for GSEA and ULM were implemented utilizing the decoupler.mt.gsea (for unweighted databases) and decoupler.mt.ulm (for the PROGENy and CollecTRI databases) functions with default parameters, respectively. Corresponding databases were identically accessed via https://omnipathdb.org/. For these analyses, we strictly retained pathways and TFs with an adjusted *P*-value < 0.05. In contrast to the AUCell selection strategy, we extracted the top 10 upregulated and top 10 downregulated pathways or TFs based on their activity scores for each test vs reference comparison. Following the removal of duplicate entries, these aggregated lists were utilized to generate final visualizations. Full raw data of all statistically significant DEGs for all 41 functionally defined cell subtypes are provided in Supplemental File 3. Cell-cell communication analysis was performed separately on inflamed lesion samples from the intestinal BD cohort and the CD cohort using CellChat v2.2.0, with permutation testing to identify significant differential interactions [38].

### Ethical statement

This study received approval from the Ethics Committee of Peking Union Medical College Hospital (protocol code: K4434; approval date: July 19, 2023). Written informed consent was obtained from all participants, with all procedures conducted according to the principles of the Declaration of Helsinki.

### Statistical analysis

All statistical analyses were conducted using Python. All tests were two-sided, with an adjusted *P* value of < 0.05 considered statistically significant unless otherwise specified. Differential expression analysis employed both the Wilcoxon test and a paired pseudobulk approach. Permutation testing was utilized for differential cell-cell communication analysis, with significance defined at *P* < 0.05.

## Results

### Single-cell transcriptomic atlas of terminal ileum tissues from patients with intestinal BD, CD, and HCs

To elucidate the cellular and molecular landscape of intestinal BD, we performed scRNA-seq on paired inflamed and adjacent histologically normal terminal ileum biopsy specimens from four patients with active intestinal BD. We subsequently integrated our dataset with a publicly available scRNA-seq dataset of terminal ileum tissues from 12 patients with CD and six HCs, facilitating a unified comparative analysis following batch effect correction (Figure 1).

**Figure 3.**
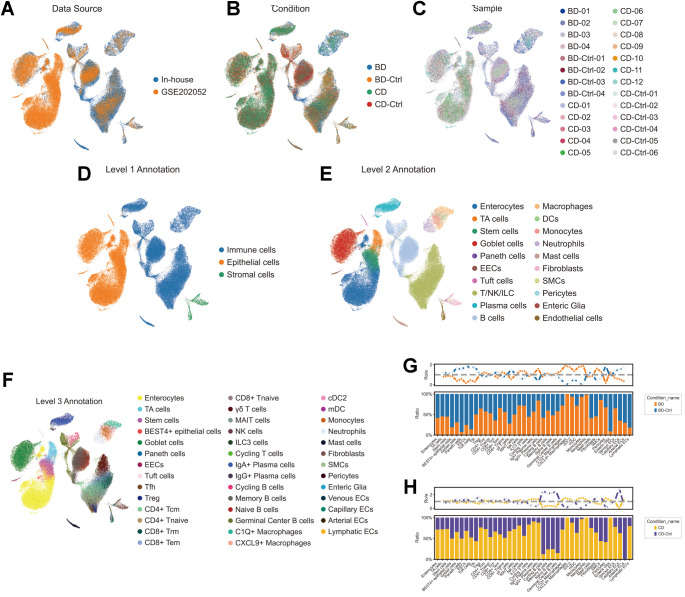
**Single-cell transcriptomic atlas of terminal ileum tissues from BD, CD, and HCs.** (A–C) UMAP embeddings of the 98,119 high-quality cells, colored by (A) data source, (B) study condition, and (C) sample origin. (D–F) UMAP visualizations illustrating the three-level hierarchical annotation strategy: (D) Level 1, (E) Level 2, and (F) Level 3. (G–H) Ro/e abundance analysis quantifying alterations in cellular composition for (G) BD lesions versus autologous non-lesional controls, and (H) CD lesions versus HCs. Abbreviations: BD, Behçet’s disease; CD, Crohn’s disease; HCs, healthy controls; Ro/e, ratio of observed/expected; UMAP, uniform manifold approximation and projection.

After rigorous quality control, a total of 98,119 high-quality cells were retained for downstream analysis. These cells originated from two independent data sources (Figure 3A), representing four study conditions (Figure 3B) and a cumulative total of 26 samples (Figure 3C). We employed a three-tier hierarchical cell annotation strategy for cell population classification: Level 1 annotation categorized all cells into three primary lineages: immune cells, epithelial cells, and stromal cells (Figure 3D); Level 2 annotation further divided these lineages into 20 distinct cell populations (Figure 3E); and the final Level 3 high-resolution annotation refined the populations into 41 functionally defined cell subtypes (Figure 3F). Analysis of the abundance of the 41 cell subtypes revealed significant alterations in the cellular composition of both BD and CD lesions, marked by an enrichment of innate immune cells, including various macrophage, neutrophil, and monocyte lineages in inflamed tissues (Figures 3G and 3H). This high-resolution atlas represents the first comprehensive characterization of the cellular composition of intestinal BD lesions, providing a foundational resource for subsequent mechanistic investigations.

### Stromal compartments exhibit robust activation and extracellular matrix (ECM) remodeling in intestinal BD with putative disease-associated features

Given that BD is classified as a systemic vasculitis, we first characterized the transcriptional and functional alterations of stromal compartments, focusing on high-resolution Level 3 subtypes, including capillary, venous, and arterial endothelial cells, as well as fibroblasts, which constitute the structural and functional core of the vascular system and tissue microenvironment.

**Figure 4.**
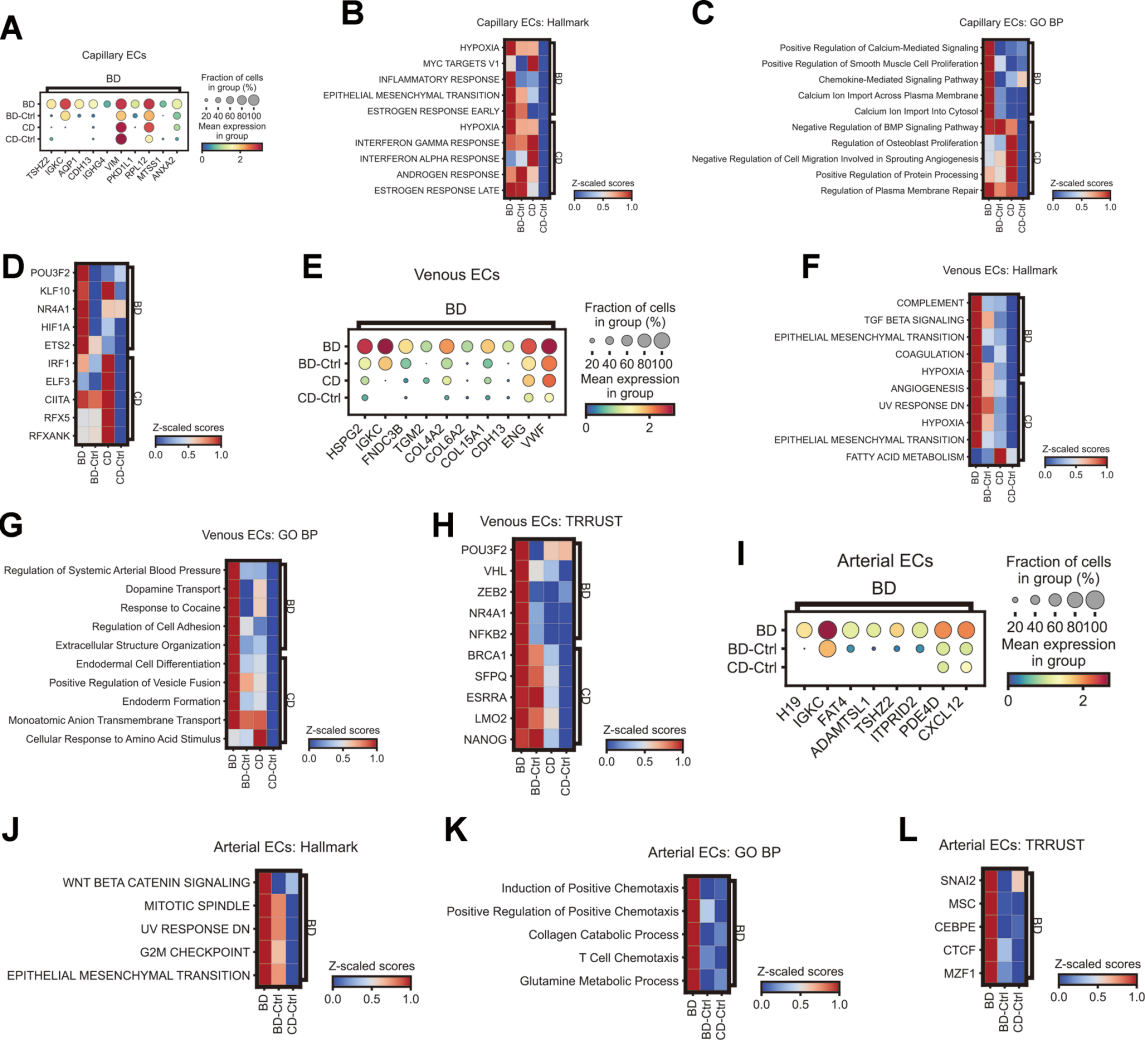
**Transcriptional signatures and functional pathway activation in stromal endothelial cell subsets.** (A–D) Capillary endothelial cells: (A) Top cell-type-specific DEGs comparing BD lesions to autologous controls; AUCell enrichment analysis of (B) MSigDB Hallmark pathways, (C) GO BP terms, and (D) TRRUST TF activities comparing BD lesions to autologous controls and CD lesions to HCs. (E–H) Venous endothelial cells: (E) Top DEGs; AUCell enrichment analysis of (F) Hallmark pathways, (G) GO BP terms, and (H) TRRUST TF activities. (I–L) Arterial endothelial cells: (I) Top DEGs; AUCell enrichment analysis of (J) Hallmark pathways, (K) GO BP terms, and (L) TRRUST TF activities. Statistical significance for single-cell DEGs was determined using the Wilcoxon rank-sum test (adjusted *P <* 0.05 and |log2FoldChange| > 1). Abbreviations: BD, Behçet’s disease; CD, Crohn’s disease; DEGs, differentially expressed genes; ECs, endothelial cells; GO BP, Gene Ontology Biological Process; HCs, healthy controls; TF, transcription factor.

In capillary endothelial cells, Wilcoxon testing identified teashirt zinc finger homeobox 2 (TSHZ2), immunoglobulin kappa constant (IGKC), aquaporin 1 (AQP1), cadherin 13 (CDH13), IGHG4, VIM, PKD1L1, RPL12, MTSS1, and ANXA2 as the ten most significantly upregulated genes in BD lesions compared to paired non-lesional controls (Figure 4A). AUCell enrichment analysis revealed distinct transcriptional profiles between the two disease states. In the BD cohort, capillary endothelial cells were significantly enriched for hypoxia, MYC targets, inflammatory responses, and epithelial-mesenchymal transition (EMT) pathways (Figure 4B). These cells also exhibited activation of calcium-mediated signaling and biological processes related to smooth muscle cell proliferation (Figure 4C). In contrast, CD samples compared to HCs showed enrichment in Hallmark pathways associated with hypoxia and interferon responses, with GO terms highlighting negative regulation of bone morphogenetic protein (BMP) signaling and angiogenesis. Furthermore, TRRUST analysis indicated that the transcriptional network in BD was driven by TFs such as POU3F2, KLF10, and HIF1A, while CD was predominantly regulated by IRF1, ELF3, and CIITA (Figure 4D).

For venous endothelial cells, the top upregulated genes in BD lesions vs autologous controls included heparan sulfate proteoglycan 2 (HSPG2), IGKC, FNDC3B, TGM2, collagen type IV alpha 2 chain (COL4A2), COL6A2, COL15A1, CDH13, ENG, and von Willebrand factor (VWF) (Figure 4E). Pathway analysis of these cells in BD demonstrated significant enrichment for complement activation, transforming growth factor beta (TGF-β) signaling, coagulation, and EMT (Figure 4F). These cells also played a role in regulating systemic arterial blood pressure and cell adhesion (Figure 4G). In contrast, venous endothelial cells in CD patients were characterized by pathways associated with angiogenesis, hypoxia, fatty acid metabolism, endodermal cell differentiation, and vesicle fusion. TF activity also varied across cohorts, with POU3F2, VHL, and ZEB2 being highly enriched in BD, whereas BRCA1, SFPQ, and ESRRA emerged as primary regulators in CD (Figure 4H).

In arterial endothelial cells, only eight genes were significantly upregulated in BD compared to self-controls, including H19, IGKC, FAT4, ADAMTSL1, TSHZ2, ITPRID2, PDE4D, and CXCL12 (Figure 4I). Due to the absence of arterial endothelial cells in the CD cohort, downstream functional enrichment was exclusively evaluated for BD. AUCell scoring indicated that arterial endothelial cells in BD were highly enriched for Wnt/β-catenin signaling, mitotic spindle assembly, and EMT pathways (Figure 4J). The predominant biological processes were heavily influenced by chemotaxis, specifically in the induction of positive chemotaxis and T cell chemotaxis (Figure 4K). Finally, TRRUST analysis identified SNAI2, MSC, CEBPE, CTCF, and MZF1 as the principal TFs driving the pathological transcriptional program of arterial endothelial cells in BD (Figure 4L).

**Figure 5.**
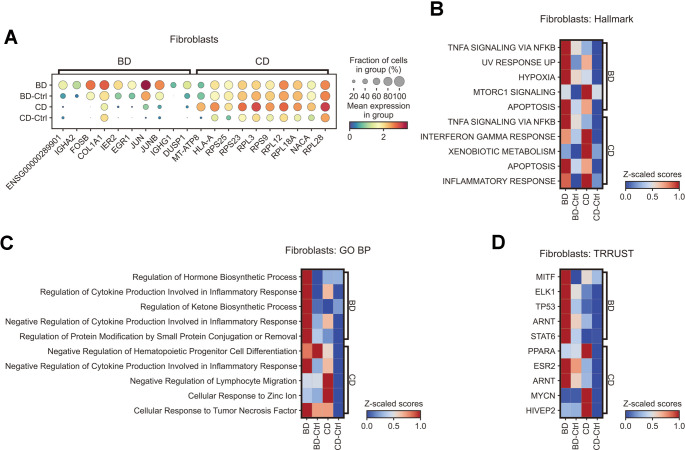
**Putative disease-associated transcriptional and functional shifts in fibroblasts.** (A) Top cell-type-specific DEGs in fibroblasts comparing BD lesions to autologous controls and CD lesions to HCs. (B) AUCell enrichment analysis of MSigDB Hallmark pathway. (C) Enrichment analysis of GO BP terms. (D) Enrichment analysis of TRRUST TF activities. Single-cell DEGs were identified via the Wilcoxon rank-sum test (adjusted *P <* 0.05 and |log2FoldChange| > 1). Abbreviations: BD, Behçet’s disease; CD, Crohn’s disease; DEGs, differentially expressed genes; GO BP, Gene Ontology Biological Process; HCs, healthy controls; TF, transcription factor.

In fibroblasts, distinct transcriptional signatures were observed. The top upregulated genes in BD lesions relative to autologous controls included ENSG00000289901, IGHA2, FOSB, COL1A1, IER2, EGR1, JUN, JUNB, IGHG1, and DUSP1. Conversely, CD samples compared to HCs were characterized by high expression of MT-ATP8, major histocompatibility complex, class I, A (HLA-A), NACA, and several ribosomal genes, including RPS25, RPS23, RPL3, RPS9, RPL12, RPL18A, and RPL28 (Figure 5A). While both diseases shared Hallmark pathways related to tumor necrosis factor alpha (TNF-α) signaling via nuclear factor kappa B (NF-κB) and apoptosis, BD fibroblasts uniquely enriched pathways associated with hypoxia, mechanistic target of rapamycin complex 1 (mTORC1) signaling, and upregulated ultraviolet (UV) responses. This contrasted with CD fibroblasts, which exhibited enrichment for interferon-γ, xenobiotic metabolism, and broad inflammatory responses (Figure 5B). Biological process analysis indicated that BD was primarily associated with the regulation of hormone and ketone biosynthesis, alongside inflammatory cytokine production. In contrast, CD was driven by cellular responses to tumor necrosis factor and zinc ions, as well as the negative regulation of lymphocyte migration and hematopoietic progenitor cell differentiation (Figure 5C). Finally, TRRUST analysis indicated that aryl hydrocarbon receptor nuclear translocator (ARNT) was a key shared TF. Despite this commonality, the BD network was predominantly regulated by melanocyte inducing transcription factor (MITF), ELK1, TP53, and signal transducer and activator of transcription 6 (STAT6), while the CD network was driven by PPARA, ESR2, MYCN, and HIVEP2 (Figure 5D).

### Myeloid phagocytes exhibit distinct disease-associated transcriptional programs in intestinal BD and CD

Next, we examined the transcriptional alterations of myeloid phagocytes, including neutrophils and macrophages, which are central mediators of innate immunity and inflammatory tissue damage in BD.

**Figure 6.**
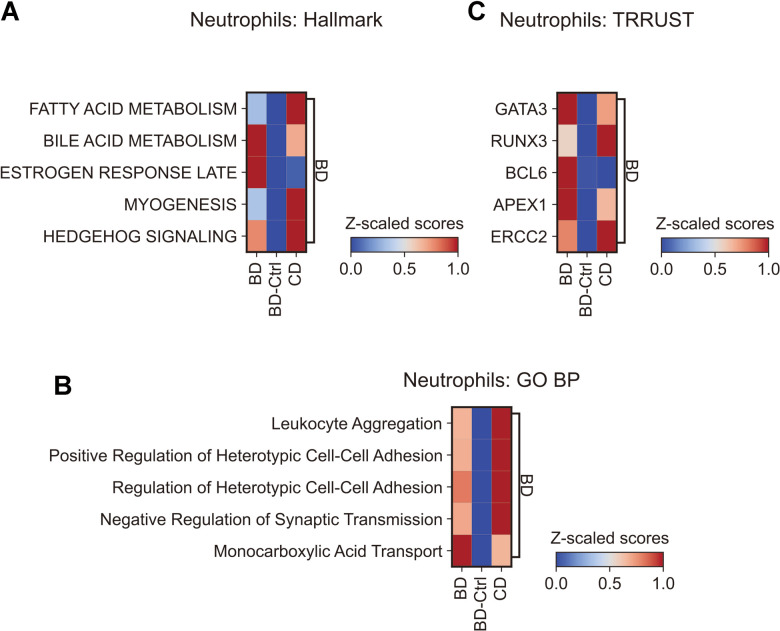
**Functional pathway activation in intestinal BD neutrophils.** (A) AUCell enrichment analysis of MSigDB Hallmark pathways comparing BD lesional neutrophils to autologous controls. (B) Enrichment analysis of GO BP terms. (C) Enrichment analysis of TRRUST TF activities. Abbreviations: BD, Behçet’s disease; GO BP, Gene Ontology Biological Process; TF, transcription factor.

Due to the insufficient number of neutrophils in the reference groups, differential gene expression analysis via the Wilcoxon test was not feasible. Specifically, only 12 neutrophil cells were present in the BD autologous controls, with none observed in HCs. The overall proportions of neutrophils captured were approximately 3.52% in inflamed BD lesions, 0.06% in paired adjacent controls, 0.71% in inflamed CD lesions, and absent (0%) in HCs (Supplemental File 4). This scarcity likely reflects the well-documented “survivor cell” bias inherent to droplet-based single-cell platforms, which limits the capture efficiency of delicate granulocytes. Consequently, downstream AUCell functional enrichment could not be evaluated for the CD cohort, and the following results should be interpreted as exploratory and supportive only: Exploratory AUCell scoring for the BD cohort suggested that lesional neutrophils might be enriched for Hallmark pathways related to fatty acid and bile acid metabolism, late estrogen responses, myogenesis, and Hedgehog signaling compared to autologous controls (Figure 6A). Furthermore, the top enriched GO biological processes included leukocyte aggregation, the general and positive regulation of heterotypic cell-cell adhesion, negative regulation of synaptic transmission, and monocarboxylic acid transport (Figure 6B). Exploratory TRRUST network analysis identified GATA3, RUNX3, BCL6, APEX1, and ERCC2 as potential primary TFs active in BD neutrophils (Figure 6C).

**Figure S2.**
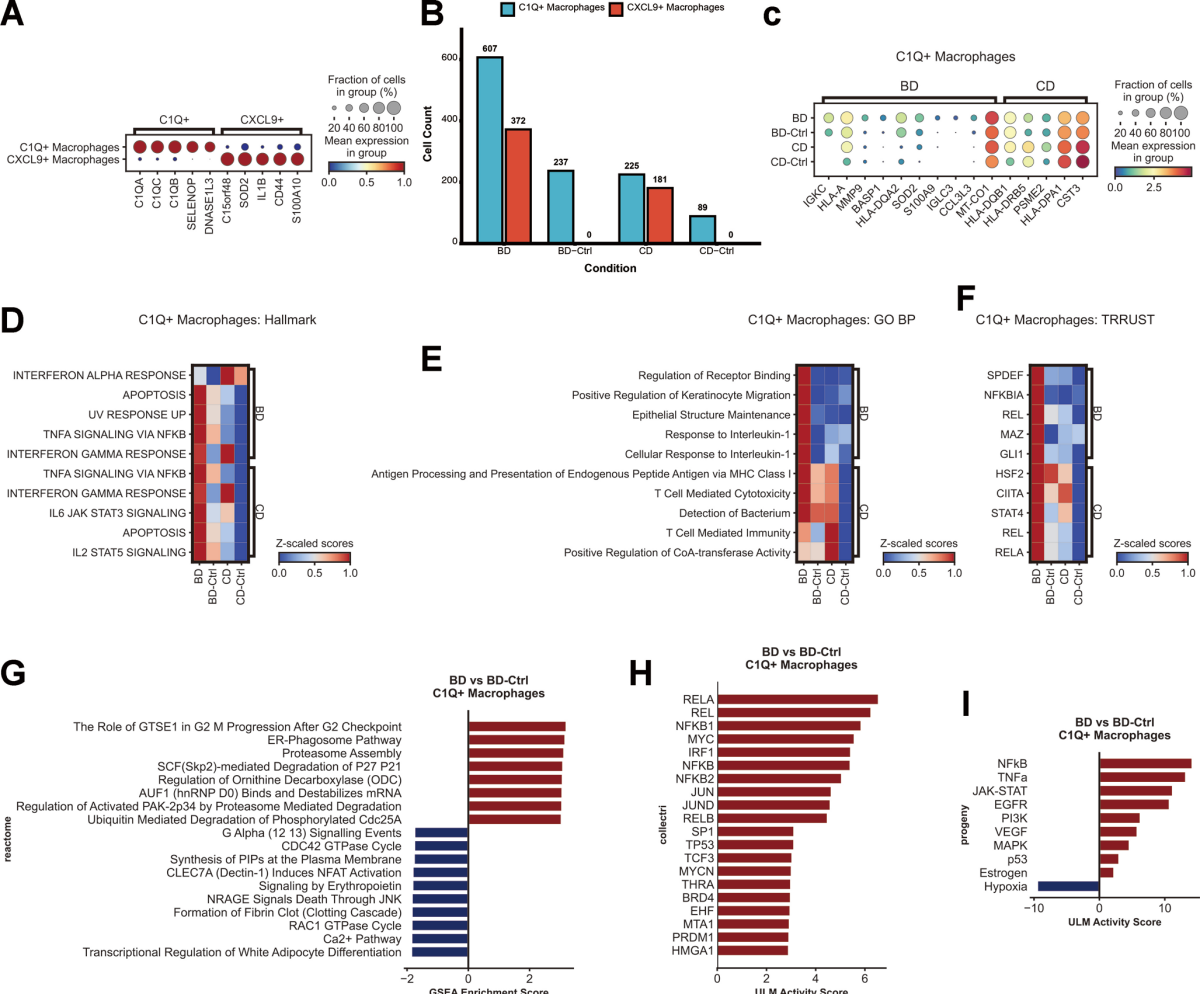
**Subclustering and transcriptomic profiling of macrophage populations.** (A–B) Macrophage subclustering showing (A) DEGs between C1Q^+^ and CXCL9^+^ subsets and (B) the distribution of these subsets across disease lesions and controls. (C–F) Single-cell level analysis of C1Q^+^ macrophages detailing (C) Wilcoxon-derived DEGs comparing BD lesions to autologous controls and CD lesions to HCs, AUCell enrichment analysis of (D) Hallmark pathways, (E) GO BP terms, and (F) TRRUST TF activities. (G–I) Downstream pathway and regulatory network analysis of BD C1Q^+^ macrophages illustrating (G) Reactome GSEA, (H) CollecTRI/ULM-inferred TF activity, and (I) PROGENy pathway activity analysis. Single-cell DEGs utilized the Wilcoxon rank-sum test (adjusted *P <* 0.05 and |log2FoldChange| > 1). Abbreviations: BD, Behçet’s disease; CD, Crohn’s disease; DEGs, differentially expressed genes; GO BP, Gene Ontology Biological Process; GSEA, Gene Set Enrichment Analysis; HCs, healthy controls; TF, transcription factor; ULM, univariate linear model.

Further subclustering of macrophages revealed two distinct populations: C1Q+ and C-X-C motif chemokine ligand 9 (CXCL9+) macrophages. Compared to the CXCL9+ subset, C1Q+ macrophages significantly overexpressed complement C1q A chain (C1QA), complement C1q C chain (C1QC), complement C1q B chain (C1QB), SELENOP, and DNASE1L3. Conversely, the top five genes upregulated in CXCL9+ macrophages were C15orf48, SOD2, IL1B, CD44, and S100A10 (Figure S2A). Notably, CXCL9+ macrophages were exclusively captured in BD and CD lesions and were entirely absent in autologous and HCs. Consequently, standard statistical testing could not be performed for this subset, and their potential roles in inflammatory pathogenesis should be interpreted with caution (Figure S2B).

In C1Q+ macrophages, Wilcoxon analysis identified IGKC, HLA-A, matrix metallopeptidase 9 (MMP9), BASP1, HLA-DQA2, SOD2, S100A9, IGLC3, CCL3L3, and MT-CO1 as the ten most significantly upregulated genes in BD compared to autologous controls. In CD, only five genes (HLA-DQB1, HLA-DRB5, PSME2, HLA-DPA1, and CST3) were significantly upregulated relative to HCs (Figure S2C). AUCell enrichment demonstrated that BD C1Q+ macrophages were characterized by interferon-α and γ responses, apoptosis, upregulated UV responses, and TNF-α signaling via NF-κB (Figure S2D), which biologically manifested as enhanced responses to interleukin-1 and maintenance of epithelial structure (Figure S2E). In contrast, CD C1Q+ macrophages exhibited robust enrichment for TNF-α via NF-κB, interferon-γ, interleukin-6–Janus kinase–signal transducer and activator of transcription 3 (IL6-JAK-STAT3), apoptosis, and interleukin-2–signal transducer and activator of transcription 5 (IL2-STAT5) signaling (Figure S2D), corresponding with enhanced major histocompatibility complex (MHC) class I antigen presentation, T cell-mediated immunity, and bacterial detection processes (Figure S2E). TRRUST network analysis further delineated distinct transcriptional regulators: BD was driven by SPDEF, NFKBIA, REL, MAZ, and GLI1, while CD was predominantly regulated by HSF2, CIITA, signal transducer and activator of transcription 4 (STAT4), REL, and RELA (Figure S2F).

Further downstream analysis using Reactome GSEA in BD C1Q+ macrophages revealed a significant upregulation of pathways related to cell cycle regulation and protein degradation. This included GTSE1, which is involved in G2/M progression, the endoplasmic reticulum (ER)-phagosome pathway, proteasome assembly, and SKP1–Cullin–F-box/S-phase kinase-associated protein 2 (SCF/Skp2)-mediated degradation. Conversely, pathways associated with G-protein signaling (Gα12/13, CDC42, RAC1), calcium pathways, and Dectin-1/nuclear factor of activated T cells (NFAT) signaling were significantly downregulated (Figure S2G). ULM activity scoring via CollecTRI confirmed a pronounced inflammatory transcriptional state in BD, characterized by the notable activation of NF-κB family members (RELA, REL, NFKB1, NFKB2), MYC, IRF1, and activator protein 1 (AP-1) components (JUN, JUND) (Figure S2H). In alignment with these findings, PROGENy analysis validated the significant hyperactivation of NF-κB, TNF-α, JAK-STAT, epidermal growth factor receptor (EGFR), phosphoinositide 3-kinase (PI3K), vascular endothelial growth factor (VEGF), mitogen-activated protein kinase (MAPK), p53, and estrogen signaling pathways in BD C1Q+ macrophages, alongside specific downregulation of hypoxia (Figure S2I).

### Epithelial barrier alterations show potential divergence between intestinal BD and CD

Intestinal epithelial cells constitute the mucosal barrier and are essential regulators of mucosal immunity. Consequently, we characterized the transcriptional alterations in two key epithelial subsets: enterocytes and goblet cells.

**Figure 7.**
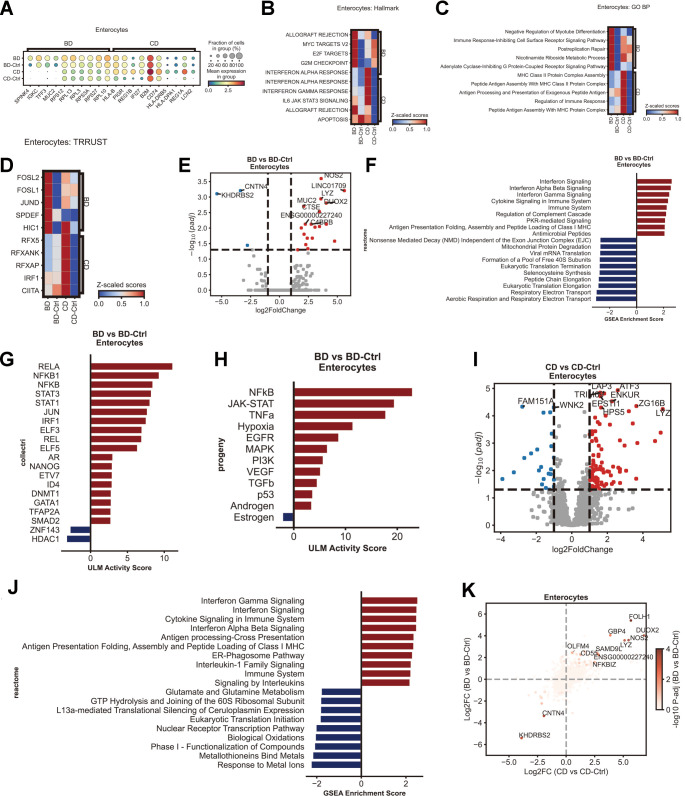
**Epithelial barrier alterations and transcriptomic profiling in enterocytes.** (A–D) Single-cell level analysis showing (A) Wilcoxon-derived DEGs comparing BD lesions to autologous controls and CD lesions to HCs, AUCell enrichment analysis of (B) Hallmark pathways, (C) GO BP terms, and (D) TRRUST TF activities. (E–H) Patient-matched pseudobulk analysis of the BD cohort: (E) DEGs, (F) Reactome GSEA, (G) CollecTRI/ ULM-inferred TF activity, and (H) PROGENy pathway activity analysis. (I–J) Pseudobulk analysis of the CD cohort showing (I) DEGs and (J) Reactome GSEA. (K) Four-quadrant cross-disease comparative analysis mapping shared and distinct enterocyte pathologies. Single-cell DEGs utilized the Wilcoxon rank-sum test (adjusted *P <* 0.05 and |log2FoldChange| > 1); pseudobulk DEGs utilized DESeq2 (adjusted *P <* 0.05 and |log2FoldChange| > 1). Abbreviations: BD, Behçet’s disease; CD, Crohn’s disease; DEGs, differentially expressed genes; GO BP, Gene Ontology Biological Process; GSEA, Gene Set Enrichment Analysis; HCs, healthy controls; MHC, major histocompatibility complex; TF, transcription factor; ULM, univariate linear model.

Wilcoxon analysis revealed distinct transcriptomic signatures in enterocytes. In BD, the most significantly upregulated genes compared to autologous controls included serine peptidase inhibitor Kazal type 4 (SPINK4), IGKC, TFF3, MUC2, and several ribosomal genes such as RPS12, RPL13, RPL3, RPS3A, RPS27, and RPL10. Conversely, CD enterocytes exhibited upregulation of components related to the MHC and antimicrobial functions compared to HCs, including HLA-B, PIGR, REG1B, IFI27, B2M, CD74, HLA-DRB5, HLA-DPA1, REG1A, and lipocalin 2 (LCN2) (Figure 7A). AUCell scoring indicated that BD enterocytes were enriched for pathways associated with allograft rejection, MYC targets V2, E2F targets, and the G2M checkpoint (Figure 7B). These pathways were primarily driven by TFs such as FOSL2, FOSL1, JUND, SPDEF, and HIC1 (Figure 7D). Biological processes in BD were dominated by the inhibition of cell surface receptor signaling and post-replication repair (Figure 7C). In contrast, CD enterocytes displayed strong enrichment for interferon-α and γ responses, IL6-JAK-STAT3 signaling, and apoptosis (Figure 7B), primarily grounded in MHC Class II assembly and antigen presentation pathways (Figure 7C). The CD network was predominantly regulated by RFX family members, IRF1, and CIITA (Figure 7D).

To rigorously validate these findings, patient-matched pseudobulk analysis was performed for the BD cohort, identifying 24 strictly filtered DEGs. Three genes were significantly downregulated, while 21 were upregulated, including folate hydrolase 1 (FOLH1), REG4, DUOX2, SPINK4, NOS2, and MUC2 (Figure 7E). The downregulated genes included CDHR1, CNTN4, and KHDRBS2. Downstream GSEA via Reactome revealed that BD enterocytes strongly upregulated interferon, general cytokine, and complement cascade signaling, alongside antimicrobial peptide activity. Conversely, pathways related to translation elongation and mitochondrial respiratory electron transport were downregulated (Figure 7F). Activity scoring corroborated this inflammatory state by highlighting the activation of TFs such as RELA, NF-κB1, signal transducer and activator of transcription 1 (STAT1), signal transducer and activator of transcription 3 (STAT3), and JUN while simultaneously suppressing ZNF143 and HDAC1 (Figure 7G). PROGENy pathway analysis confirmed significant activation of NF-κB, JAK-STAT, TNF-α, hypoxia, and EGFR signaling in BD enterocytes, alongside downregulation of estrogen signaling (Figure 7H).

Pseudobulk analysis of the CD cohort identified 153 significantly DEGs compared to HCs (Figure 7I). Similar to BD, Reactome enrichment demonstrated upregulation of interferon, interleukin, and antigen presentation pathways in CD. However, CD was uniquely characterized by downregulation of glutamate metabolism, translation initiation, and biological oxidation processes (Figure 7J). A four-quadrant cross-disease comparison highlighted the intersection of these pathologies. A core shared inflammatory module was consistently upregulated in both BD and CD, encompassing FOLH1, GBP4, DUOX2, NOS2, LYZ, SAMD9L, ENSG00000227240, and NFKBIZ. Additionally, CNTN4 and KHDRBS2 were downregulated in both diseases. Notably, OLFM4 and CD55 were significantly upregulated exclusively in BD, suggesting potential differences in the enterocyte transcriptomic profile between BD and CD within this study cohort (Figure 7K).

**Figure 8.**
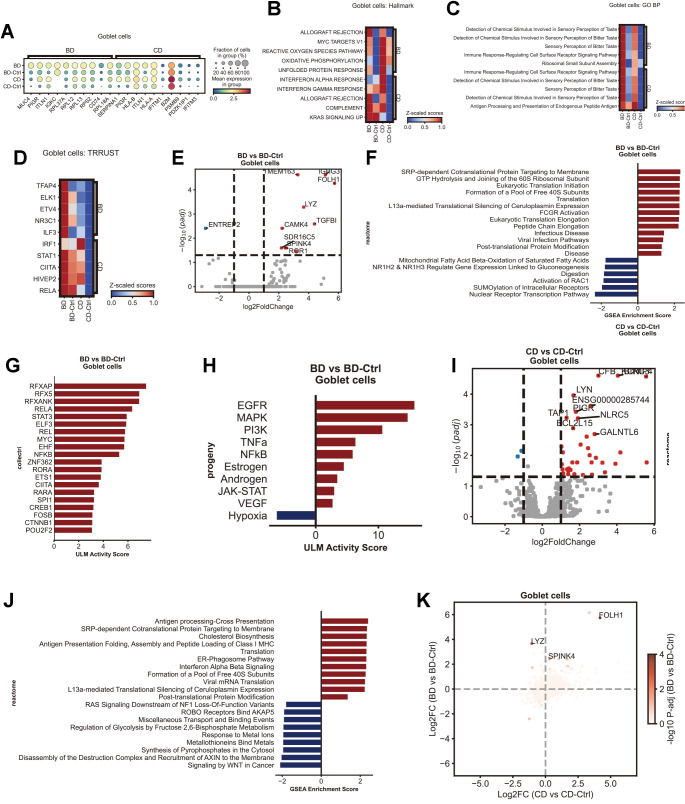
**Putative disease-divergent transcriptional alterations in goblet cells.** (A–D) Single-cell level profiling detailing (A) Wilcoxon-derived DEGs comparing BD lesions to autologous controls and CD lesions to HCs, AUCell enrichment analysis of (B) Hallmark pathways, (C) GO BP terms, and (D) TRRUST TF activities. (E–H) Patient-matched pseudobulk analysis of the BD cohort illustrating (E) DEGs, (F) Reactome GSEA, (G) CollecTRI/ULM-inferred TF activity, and (H) PROGENy pathway activity analysis. (I–J) Pseudobulk analysis of the CD cohort mapping (I) DEGs and (J) Reactome GSEA. (K) Four-quadrant cross-disease integration highlighting globally shared versus disease-specific markers. Single-cell DEGs utilized the Wilcoxon rank-sum test (adjusted *P <* 0.05 and |log2FoldChange| > 1); pseudobulk DEGs utilized DESeq2 (adjusted *P <* 0.05 and |log2FoldChange| > 1). Abbreviations: BD, Behçet’s disease; CD, Crohn’s disease; DEGs, differentially expressed genes; GO BP, Gene Ontology Biological Process; GSEA, Gene Set Enrichment Analysis; HCs, healthy controls; TF, transcription factor; ULM, univariate linear model.

In goblet cells, Wilcoxon analysis identified distinct transcriptional alterations between the two diseases. BD lesions exhibited significant upregulation of MUC4, PIGR, ITLN1, IGKC, CD74, and multiple ribosomal genes, including RPL37A, RPL12, RPL13, RPS2, and RPL18A. In contrast, CD cells were characterized by high expression of SERPINA1, PIGR, HLA-B, ITLN1, HLA-A, IFITM1, B2M, PSMB9, PDZK1IP1, and IFITM3 relative to HCs (Figure 8A). AUCell enrichment indicated that BD goblet cells were primarily driven by pathways associated with allograft rejection, MYC targets V1, reactive oxygen species, oxidative phosphorylation, and unfolded protein responses (Figure 8B). Biological processes in BD highlighted sensory perception of taste and bitter stimuli, immune receptor signaling, and ribosomal small subunit assembly (Figure 8C). Conversely, CD goblet cells were dominated by interferon responses, complement activation, and antigen presentation, while sharing enrichments for allograft rejection and immune receptor signaling with BD (Figures 8B and 8C). TRRUST network analysis indicated that BD was uniquely regulated by TFs such as TFAP4, ELK1, ETV4, NR3C1, and ILF3, whereas the CD transcriptional program was driven by IRF1, STAT1, CIITA, HIVEP2, and RELA (Figure 8D).

Patient-matched pseudobulk analysis was conducted on the BD cohort to rigorously validate these findings, identifying 12 strictly filtered DEGs. Specifically, ENTREP2 was significantly downregulated while 11 genes were upregulated, including FOLH1, IGHG3, ST3GAL4, TGFBI, LYZ, TMEM163, ROR2, ROR1, SPINK4, CAMK4, and SDR16C5 (Figure 8E). Downstream Reactome enrichment demonstrated that BD goblet cells broadly upregulated translation-related processes, viral infection pathways, and Fc gamma receptor (FCGR) activation. Conversely, pathways associated with mitochondrial fatty acid β-oxidation, digestion, and nuclear receptor transcription were suppressed (Figure 8F). Activity scoring corroborated this active inflammatory and translational state, showing significant activation of TFs including RFX family members, RELA, STAT3, and NF-κB (Figure 8G). PROGENy pathway analysis confirmed the upregulation of EGFR, MAPK, PI3K, TNF-α, NF-κB, and JAK-STAT signaling alongside notable downregulation of hypoxia in BD (Figure 8H).

Pseudobulk analysis of the CD cohort yielded 44 DEGs compared to HCs (Figure 8I). Reactome enrichment in CD mirrored BD regarding the upregulation of translational machinery and viral mRNA processing; however, it was uniquely characterized by enhanced Class I MHC antigen presentation and cholesterol biosynthesis, alongside downregulation of metal ion responses, glycolysis regulation, and Wnt signaling (Figure 8J). A four-quadrant cross-disease comparison highlighted the intersection of these goblet cell pathologies. Notably, only FOLH1 was significantly upregulated across both BD and CD, while SPINK4 and LYZ were significantly upregulated exclusively in BD without significant analogous changes in the CD cohort, underscoring putative disease-associated transcriptomic shifts in intestinal goblet cells (Figure 8K).

### Dysregulated cell-cell communication networks identify stromal-immune crosstalk as a potential driver of intestinal BD

To understand how distinct cell types coordinate to drive intestinal inflammation in BD, we mapped cell-cell communication networks in intestinal BD and CD and compared their signaling patterns.

**Figure 9.**
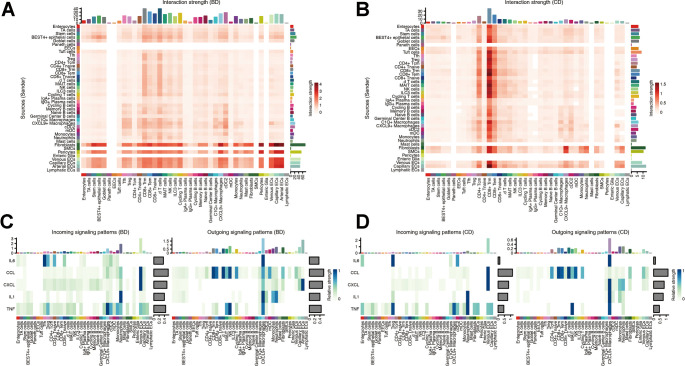
**Cell-cell communication networks in intestinal BD and CD.** (A–B) Heatmaps of cell-cell interaction strength across 41 cell types in (A) intestinal BD and (B) CD. (C–D) Heatmaps of incoming and outgoing signaling patterns of several specific pathways across 41 cell types in (C) intestinal BD and (D) CD. Cell-cell communication analysis was performed separately in inflamed lesions of intestinal BD and CD, with CellChat permutation testing (*P <* 0.05) to identify significant interactions within each disease cohort. Abbreviations: B, B lymphocyte; BD, Behçet’s disease; CD, Crohn’s disease; CD, cluster of differentiation; cDC2, conventional dendritic cell type 2; CXCL, C-X-C motif chemokine ligand; EC, endothelial cell; EECs, enteroendocrine cells; IgA, immunoglobulin A; IgG, immunoglobulin G; ILC3, innate lymphoid cell type 3; MAIT, mucosal-associated invariant T; mDC, myeloid dendritic cell; NK, natural killer; SMCs, smooth muscle cells; T, T lymphocyte; TA, transit amplifying; Tcm, central memory T; Tem, effector memory T; Tfh, T follicular helper; Tna, naive T; Treg, regulatory T; Trm, tissue-resident memory T.

Analysis of the inferred strength of signaling interactions suggested that fibroblasts, pericytes, venous endothelial cells, capillary endothelial cells, and arterial endothelial cells were the predicted dominant signaling senders in intestinal BD (Figure 9A), aligning with their central role in BD pathogenesis. In contrast, CD demonstrated a potentially distinct communication pattern, with CD8+ tissue-resident memory T (Trm) cells, CD8+ effector memory T (Tem) cells, and CD4+ central memory T (Tcm) cells serving as the major signaling receivers (Figure 9B).

Further dissection of specific signaling pathways indicated the presence of potentially disease-associated active axes. For predicted outgoing signaling in intestinal BD, the IL6 pathway appeared strongest in the sparse CXCL9+ macrophages, while the C-C motif chemokine ligand (CCL) pathway was dominant in gamma-delta T cells. The C-X-C motif chemokine ligand (CXCL) pathway peaked in arterial endothelial cells, and the TNF pathway was strongest in natural killer (NK) cells. Regarding inferred incoming signaling, the IL6 pathway demonstrated the greatest strength in T follicular helper (Tfh) cells, whereas CCL and CXCL chemokine pathways were predominant in venous endothelial cells. Additionally, the IL1 pathway was strongest in neutrophils, and the TNF pathway was dominant in CXCL9+ macrophages (Figure 9C).

In CD, a distinct pattern of outgoing signaling was observed, with CCL signaling predicted to be dominant in gamma-delta T cells, alongside inferred IL6, CXCL, and IL1 signaling from CXCL9+ macrophages, and TNF signaling from both mucosal-associated invariant T (MAIT) cells and CXCL9+ macrophages. Correspondingly, for incoming signaling, CCL and CXCL signaling were dominant in venous endothelial cells, while IL1 and TNF signaling were strongest in neutrophils, and IL6 signaling was most pronounced in regulatory T (Treg) cells (Figure 9D).

Given the critical role of endothelial cell-neutrophil crosstalk in the pathogenesis of vasculitis and BD, we conducted a detailed analysis of the primary ligand-receptor pairs between distinct subsets of endothelial cells (source) and neutrophils (target) in intestinal BD (Table 1). The collagen pathways exhibited the highest interaction probabilities. The COL4A2-CD44 and COL4A1-CD44 pairs from capillary, arterial, and venous endothelial cells ranked among the top six interactions. Following these were pairs associated with the laminin pathway, specifically LAMA4-CD44 from arterial endothelial cells and LAMC1-CD44 from capillary endothelial cells. These pairs are predicted to mediate ECM-receptor interactions, which may play a role in regulating leukocyte adhesion, migration, and activation. Additionally, we identified the computationally significant ANXA1-FPR1 and ANXA1-FPR2 pairs from the annexin pathway across all three endothelial cell subsets, which are predicted to modulate neutrophil activation and inflammatory responses through secreted signaling. Furthermore, prominent APP-SORL1 pairs from the amyloid precursor protein pathway were detected between arterial, capillary, and venous endothelial cells and neutrophils, suggesting their role in mediating essential cell-cell contact signaling.

**Table 1:** The top 15 ligand-receptor interactions between distinct endothelial cell subsets and neutrophils in intestinal Behçet’s disease

**Source**	**Target**	**Ligand**	**Receptor**	**Probability**	**Evidence**
Capillary ECs		COL4A2	CD44	0.12897504	KEGG: hsa04512
Arterial ECs		COL4A2	CD44	0.120088646	KEGG: hsa04512
Capillary ECs		COL4A1	CD44	0.114846226	KEGG: hsa04512
Venous ECs		COL4A2	CD44	0.11229274	KEGG: hsa04512
Arterial ECs		COL4A1	CD44	0.110780683	KEGG: hsa04512
Venous ECs		COL4A1	CD44	0.085027209	KEGG: hsa04512
Venous ECs		ANXA1	FPR1	0.083500037	PMID: 23230437
Capillary ECs	Neutrophils	ANXA1	FPR1	0.068399876	PMID: 23230437
Arterial ECs		ANXA1	FPR1	0.061433692	PMID: 23230437
Arterial ECs		APP	SORL1	0.059092837	PMID: 24523320
Venous ECs		ANXA1	FPR2	0.055885221	PMID: 23230437
Capillary ECs		APP	SORL1	0.05324904	PMID: 24523320
Arterial ECs		LAMA4	CD44	0.052389644	KEGG: hsa04512
Capillary ECs		LAMC1	CD44	0.051759163	KEGG: hsa04512
Venous ECs		APP	SORL1	0.051490229	PMID: 24523320

Abbreviations: ANXA1, annexin A1; APP, amyloid beta precursor protein; CD44, cluster of differentiation 44; COL4A1, collagen type IV alpha 1 chain; COL4A2, collagen type IV alpha 2 chain; ECs, endothelial cells; FPR1, formyl peptide receptor 1; FPR2, formyl peptide receptor 2; KEGG, Kyoto Encyclopedia of Genes and Genomes; LAMA4, laminin subunit alpha 4; LAMC1, laminin subunit gamma 1; PMID, PubMed identifier; SORL1, sortilin-related receptor 1.

## Discussion

This study presents the first high-resolution single-cell transcriptomic atlas of intestinal BD, a rare and refractory inflammatory vasculitis, and performs an integrative comparative analysis with CD, a clinically overlapping disorder. Our findings delineate both shared inflammatory programs and potentially distinct disease-associated cellular and molecular signatures between intestinal BD and CD, enhancing our understanding of their unique pathogenic mechanisms and underscoring the utility of single-cell omics in elucidating rare human diseases.

A central, novel finding of our study is the robust activation of stromal compartments in intestinal BD. Specifically, capillary, venous, and arterial endothelial cells, along with fibroblasts, emerged as the predominant signaling senders in BD lesions. These cells exhibited significant upregulation of EMT, hypoxia, complement activation, and chemotaxis, consistent with the core vasculitic nature of BD [39–42]. In contrast, CD displayed only minimal stromal alterations, with predominant transcriptional disruption in epithelial cells and interferon-driven immune activation. These findings provide new insights into a longstanding clinical ambiguity: while intestinal BD and CD share overlapping ileocecal ulcer phenotypes, our parallel intra-disease comparative analysis identified potentially distinct transcriptomic programs between the two disorders [11]. Our data suggest that intestinal BD may primarily be driven by stromal-vascular alterations, with secondary epithelial and immune activation, whereas CD is characterized by dominant epithelial barrier dysfunction and adaptive immune dysregulation within this study cohort. This distinction carries significant implications for diagnostic biomarker development and therapeutic selection, as current inflammatory bowel disease therapies primarily target epithelial and immune pathways [43, 44], which may be less effective for the stromal-vascular pathology of intestinal BD.

Our comparative analysis of the epithelial compartment further emphasizes these distinct disease trajectories. Enterocytes and goblet cells in both diseases shared a core inflammatory module, including upregulation of FOLH1. However, BD epithelial cells uniquely overexpressed specific antimicrobial and structural genes such as SPINK4, LYZ, and CD55. Additionally, BD enterocytes showed unique enrichment for allograft rejection and MYC target pathways driven by specific TFs like FOSL2 and JUND, contrasting sharply with the robust MHC Class II assembly and antigen presentation observed in CD enterocytes.

Our study also sheds light on innate immune dysregulation in intestinal BD. Neutrophil hyperreactivity is a well-documented hallmark of BD; however, its role in local intestinal inflammation has remained unclear [45, 46]. Due to low cell counts in control tissues, traditional differential expression analysis was unfeasible for neutrophils. Nevertheless, exploratory functional scoring suggested that lesional BD neutrophils might be enriched for fatty acid metabolism and leukocyte aggregation. Our cell-cell communication analysis further indicated strong IL1, TNF, and chemokine signaling axes targeting neutrophils, along with significant endothelial cell-neutrophil crosstalk mediated by collagen-CD44 and laminin-CD44 interactions. CD44, a key adhesion molecule, regulates leukocyte trafficking and activation in inflammatory disorders [47, 48]. Based on these exploratory observations, we hypothesize that targeting CD44-mediated ECM-receptor interactions may constitute a viable strategy to inhibit neutrophil recruitment and tissue damage in intestinal BD, which requires further experimental validation [49, 50]. Additionally, we observed substantial annexin and amyloid precursor protein (APP) pathway signaling directed at neutrophils from endothelial sources, further supporting the existence of a complex stromal-immune regulatory network.

From a therapeutic perspective, our findings align with the clinical efficacy of anti-TNF agents in intestinal BD [51] and provide a potential mechanistic rationale for targeting additional pathways. The robust IL6 pathway activity inferred from our cell-cell communication analysis offers a preliminary preclinical rationale for exploring IL6 receptor blockade (e.g., tocilizumab), which has shown promise in small clinical studies of BD [52, 53]. The widespread activation of JAK-STAT signaling across multiple cell types suggests that JAK inhibitors may be effective for intestinal BD, as demonstrated in other systemic vasculitides and inflammatory bowel diseases [54–56]. Notably, the significant stromal remodeling and ECM-receptor crosstalk we identified highlight the potential for targeting stromal activation and leukocyte adhesion, which are not addressed by current cytokine-targeted therapies [57, 58].

Furthermore, our results underscore the profound tissue specificity of intestinal BD. While high-quality datasets exist for peripheral blood mononuclear cells [7] and aortic tissue [8, 9], our study reveals a localized pathogenic program in the terminal ileum that is not fully captured in peripheral tissues or other vascular beds. This organ tropism emphasizes the necessity of studying the actual site of disease to comprehend the ”vasculitis-like” nature of intestinal involvement.

It is important to note that the comparative analysis between BD and CD in this study was based on parallel intra-disease comparisons (BD: inflamed vs. adjacent non-lesional tissues; CD: inflamed vs. healthy control tissues), rather than a direct quantitative comparison between the two disease cohorts. These differing control baselines imply that the findings cannot be directly interpreted as “disease-specific” or “unique” features of BD, but rather as distinct transcriptomic alteration patterns observed within their respective intra-disease contexts.

We acknowledge several limitations in this study. First, given the rarity of intestinal BD, the sample size of enrolled patients was relatively small. Second, regarding the control cohorts, the use of paired histologically normal adjacent tissues for BD patients (due to ethical and logistical constraints) introduces a potential field effect, which may lead to an underestimation of transcriptomic differences. This inconsistency in control types compared to the public CD cohort (independent age- and sex-matched healthy individuals), along with age differences between cohorts and the potential influence of ongoing medications, constitutes an inherent limitation for cross-disease integrated analysis. Third, our study did not include intestinal tuberculosis samples for integrated comparative analysis, although ileocecal ulceration in clinical practice can also be caused by this condition [59], which may restrict the generalizability of our findings for the differential diagnosis of ileocecal ulcers. Methodologically, the Wilcoxon rank-sum test at the single-cell level carries a risk of pseudoreplication; therefore, these relevant results should be considered supportive, whereas the core findings backed by patient-matched pseudobulk analysis are more robust. Finally, due to the limited terminal ileum tissue obtained via endoscopic biopsy, there was no surplus for additional experimental validation. Consequently, all candidate biomarkers, therapeutic targets, and inferred cell-cell interactions remain hypothesis-generating without orthogonal validation at the protein or spatial level. The current absence of a widely accepted cellular or animal model for intestinal BD further limits in-depth validation of the identified pathogenic mechanisms [60, 61].

For future research, we will continue to recruit patients and expand the sample cohort to validate our findings. Our team is also actively working to establish a stable disease model for intestinal BD to verify the function of candidate pathogenic axes and therapeutic targets. Furthermore, integrating spatial multi-omics technologies in subsequent studies will help elucidate the in situ spatial organization of pathogenic cell populations and their interactions within the intestinal microenvironment [62, 63], thereby refining our understanding of intestinal BD pathogenesis.

## Conclusion

In conclusion, our study presents the first high-resolution single-cell transcriptomic atlas of intestinal BD, characterizing its potential pathogenic landscape and stromal-immune crosstalk networks that exhibit notable differences from CD within this parallel intra-disease comparison framework. These findings address a critical gap in our understanding of intestinal BD pathogenesis, identify potential diagnostic biomarkers and therapeutic targets for further validation, and highlight the potential of integrative single-cell omics in advancing the study of rare human inflammatory diseases.

## Supplemental data

**Supplemental File 1:** **Intestinal BD scRNA-seq Sample Level Quality Control Metrics** – this file provides complete sample-level quality control (QC) metrics for the single-cell RNA sequencing (scRNA-seq) dataset of intestinal Behçet’s disease (BD) in this study, with 3 independent sheets covering the full analytical workflow: *CellRanger_Per_Sample_QC_Metric* for raw sequencing and genome alignment QC metrics generated by CellRanger v7.1.0, *CellBender_Per_Sample_QC_Metric* for per-sample QC data after ambient RNA removal via CellBender v0.3.2, and *Scanpy_scDblFinder_QC_Stepwise* for stepwise cell count tracking across the entire QC filtering process.

**Supplemental File 2:** **QC Passed Cells for Final Analysis** – this archive provides comprehensive cell-level quality control (QC) data for all final analysis-qualified cells from the single-cell RNA sequencing (scRNA-seq) dataset of intestinal Behçet’s disease (BD) in this study, with full QC metrics organized across 6 hierarchical levels: data source, experimental condition, sample origin, and Level 1–3 cell annotation. The complete metrics contained in this archive include gene detection statistics, Unique Molecular Identifier (UMI) counts, mitochondrial and ribosomal gene proportion statistics, and doublet scores for each qualified cell, to ensure full transparency and reproducibility of the downstream bioinformatic analysis.

**Supplemental File 3:** **All Statistically Significant DEGs** – this compressed archive provides full raw data of all statistically significant differentially expressed gene (DEG) results from the single-cell RNA sequencing (scRNA-seq) dataset of intestinal Behçet’s disease (BD) and Crohn’s disease (CD) in this study, with DEG analyses performed for all 41 functionally annotated cell subtypes via two complementary approaches: the cell-level Wilcoxon rank-sum test and the patient-matched pseudobulk DESeq2 analysis. The complete results contained in this archive cover two independent pairwise comparisons: BD inflamed lesions versus paired autologous non-lesional control tissues, and CD inflamed lesions versus healthy control tissues, to ensure full transparency and reproducibility of the transcriptomic and comparative analyses in this work.

**Supplemental File 4:** **Intestinal BD scRNA-seq Level3 Cell Composition Count and Percentage** – this file provides complete cellular composition metrics for the single-cell RNA sequencing (scRNA-seq) dataset of intestinal Behçet’s disease (BD) and Crohn’s disease (CD) in this study, covering all 41 functionally defined cell subtypes from the Level 3 hierarchical cell annotation. The file contains two core datasets across 4 pre-specified experimental conditions: BD (inflamed lesions of intestinal BD), BD-Ctrl (paired autologous non-lesional control tissues of BD), CD (inflamed lesions of Crohn’s disease), and CD-Ctrl (healthy control tissues for the CD cohort): absolute cell count metrics for each cell subtype, and relative cell abundance percentage normalized to total cells per condition, to ensure full transparency and reproducibility of the cellular composition analysis and cross-condition comparative analyses in this work.

**Table S1:** Clinical characteristics of four patients with active intestinal Behçet’s disease

**Variable**	**Patient 1**	**Patient 2**	**Patient 3**	**Patient 4**
**Demographics**				
Gender	Male	Female	Male	Male
Age (years)	56	35	31	42
Ethnicity	Han Chinese	Han Chinese	Han Chinese	Han Chinese
**Disease Activity**				
BDCAF score	2	2	2	2
**Active clinical features at the time of sampling**				
Oral ulcers	Present	Present	Present	Present
Genital ulcers	Absent	Absent	Absent	Absent
Skin involvement	Absent	Absent	Absent	Absent
Cardiovascular involvement	Absent	Absent	Absent	Absent
Intestinal involvement	Present	Present	Present	Present
Pathergy reaction	Negative	Negative	Negative	Negative
Arthritis	Absent	Absent	Absent	Absent
Neurological involvement	Absent	Absent	Absent	Absent
Epididymitis	Absent	Absent	Absent	Absent
Hematologic involvement	Absent	Absent	Absent	Absent
**Endoscopic Image**	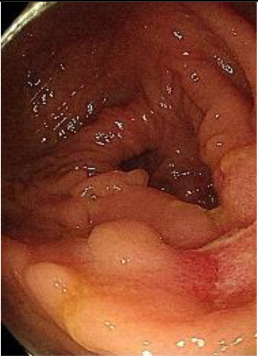	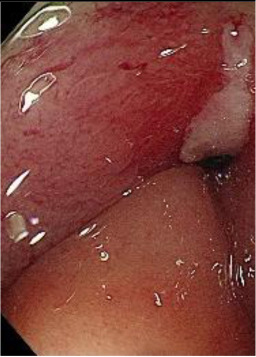	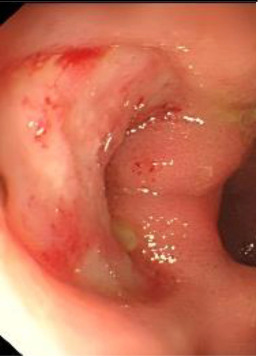	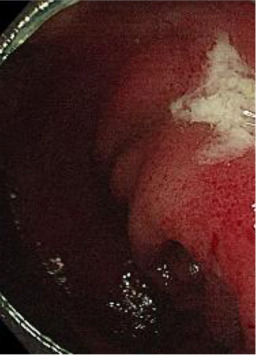
**Core Intestinal Maintenance Therapy**	Mesalazine, Sulfasalazine, Probiotics	Mesalazine	Mesalazine, PPI	No long-term maintenance therapy recorded
**Immunomodulatory/Biologic/Glucocorticoid Therapy**	Oral glucocorticoid, Thalidomide	Adalimumab	None recorded	No long-term use; single-dose dexamethasone for imaging preparation only
**Pre-Biopsy Medication Timeline**	Initial therapy Jun 2024; regimen maintained at Mar 21, 2025 pre-biopsy visit	Regimen initiated before Oct 17, 2024; maintained at Apr 2, 2025 pre-biopsy visit	Regimen initiated Apr 2024; maintained at Apr 12, 2025 pre-biopsy visit	No regular long-term medication before Jun 6, 2025 pre-biopsy visit

Specific details regarding medication doses, treatment durations, and the exact timing of the last dose before biopsy were unavailable due to limitations in patient recall. Abbreviations: BDCAF, Behçet’s Disease Current Activity Form; PPI, proton pump inhibitor.

## Data Availability

The scRNA-seq datasets generated in this study, derived from paired inflamed and adjacent histologically normal terminal ileum biopsy specimens from 4 patients with active intestinal BD, are available from the corresponding author upon reasonable request. The single-cell transcriptomics datasets of terminal ileum tissues from 12 patients with CD and 6 HCs used in this study are publicly accessible in the Gene Expression Omnibus database under accession number GSE202052 (https://www.ncbi.nlm.nih.gov/geo/query/acc.cgi?acc=GSE202052).
